# Mandibular Canine Dimorphism in Establishing Sex Identity in the Lebanese Population

**DOI:** 10.1155/2014/235204

**Published:** 2014-02-10

**Authors:** Fouad Ayoub, Loubna Shamseddine, Mohamad Rifai, Antoine Cassia, Randa Diab, Ibrahim Zaarour, Maria Saadeh, Georges Rouhana

**Affiliations:** ^1^Department of Basic Sciences, School of Dentistry, Lebanese University, Beirut, Lebanon; ^2^Department of Prosthodontics, School of Dentistry, Lebanese University, Beirut, Lebanon; ^3^Department of Periodontology, School of Dentistry, Lebanese University, Beirut, Lebanon; ^4^Department of Oral Pathology, School of Dentistry, Lebanese University, Beirut, Lebanon; ^5^Department of Orthodontics, School of Dentistry, Lebanese University, Beirut, Lebanon; ^6^Division of Orthodontics and Dentofacial Orthopedics, American University of Beirut Medical Center, Lebanon; ^7^Department of Radiology, Faculty of Medical Sciences, Lebanese University, Beirut, Lebanon

## Abstract

*Background and Objective.* In forensic investigations, mandibular canines provide excellent materials to identify gender since they are more likely to survive disasters. The objective of this study was to investigate gender dimorphism by comparing the mesiodistal width of mandibular permanent canines and intercanine distance in a group of Lebanese population. *Methods.* Participants consisted of undergraduate students from the School of Dentistry, Lebanese University, for two academic years who fulfilled the inclusion criteria. Canine widths and intercanine distance were measured by one operator directly on dental casts using a digital caliper. *Results.* One hundred thirty-three Lebanese dental students (54 males and 69 females) aged 18–25 were included in the study. The intercanine distance was significantly greater in males (*P* value < 0.0001). The right and the left canine widths were significantly greater in males than in females (*P* value < 0.0001). However, no significant difference was found between left and right canines for males (*P* value > 0.05) and females (*P* value > 0.05). The mean width of canine was greater than 7.188 mm for males. *Conclusion.* The parameters measured in the present study are of great help in sex identification in forensic investigations in the Lebanese adult population.

## 1. Introduction

Although the degree of dimorphism varies within different populations, sexual variation in the human skeleton and dentition is of great concern for anthropologists [1–4]. Sexual dimorphism refers to differences in size and form between males and females that can be applied to dental identification. In contemporary human populations, males have larger tooth crowns than females [5–8]. Consequently, tooth size standards based on odontometric investigations could be a reliable method in sex determination when limited skeletal remnants are recovered or are confusing [9–11].

Mandibular canines are considered as the “key teeth” for personal identification since they are the last teeth to be extracted with respect to age, they are less affected than other teeth by oral diseases, and are better likely to survive severe trauma such as air disaster, hurricane, or fire [2]. Recent studies have shown that the most dimorphic tooth is the mandibular canine and it can be of immense medicolegal use in identification [3, 5, 8]. The measure of the coronal tissue proportions of permanent mandibular canines suggests that males have heavier teeth and more dentin than their female counterparts [1]. The mesiodistal measure of mandibular canine and the mandibular intercanine distance are a simple inexpensive method that could be useful in forensic odontology establishing sex identity, and is of particular interest in adults aged 18–25 years [9, 10, 12–15].

No mandibular canine measurements using mesiodistal width and intercanine arch distance were available for Lebanese adults prior to this paper.

The aim of this study was to establish mandibular canine width and intercanine dimensions in a group of Lebanese adults and to compare these measurements between males and females for forensic purposes.

## 2. Materials and Methods

### 2.1. Study Population

The study was conducted at the Lebanese University School of Dentistry, Beirut, Lebanon. All undergraduate students for the academic years 2006-2007 and 2011-2012 were invited to participate after fulfilling the following inclusion criteria:

- parents and grandparents of Lebanese origin;
- age from 18 to 25 years;
- presence of the mandibular canines;
- absence of morphological tooth abnormalities;
- absence of crowding or spacing in the anterior teeth;
- absence of carious lesions or fillings in the interproximal aspects of the mandibular canines;
- absence of severe abrasion, attrition, or fracture on the involved teeth;
- healthy periodontal status;
- normal overjet and overbite;
- normal molar and canine relationship;
- no previous or current orthodontic treatment.

The age which was limited to the range of 18 to 25 years was selected since abrasion is minor in this age category.

One hundred twenty-three students (54 males and 69 females) met the inclusion criteria and were included in the study. Informed consent was obtained from all the participants.

### 2.2. Measurements

Mandibular dental impressions were made using polysiloxane in a double mixture base (Zetaplus, Indurent Oranwash L, Zhermack, Italy) and casts were poured in type 3 orthodontic dental stone (Elite Ortho, Zhermack, Italy) within half an hour. Impressions were repeated when the casts did not allow accurate measurements of the teeth.

Measuring was performed as described by Hunter and Priest [16]. One calibrated operator took all the measurements in a well-illuminated room using a digital caliper with an accuracy of 0.02 millimeter (Digimatic caliper, Mitutoyo, UK).

The following measurements were performed and recorded:

- mesiodistal mandibular canine width: defined as the greatest distance between the proximal surfaces of the crown. Both right and left canines were measured;
- intercanine distance: defined as the linear distance between the tips of the two mandibular canines;
- sexual dimorphism in right and left mandibular canines: calculated according to the formula given by Garn et al. [17] as follows:
   Sexual Dimorphism in percentage (%) = (*X*_*m*_ − 1)/*X*_*f*_ × 100, where *X*_*m*_ is the mean value for males and *X*_*f*_ is the mean value for females.

### 2.3. Statistical Analysis

Variables were tested for normal distribution using Kolmogorov-Smirnov test and for equality of variance using Levene's test. Data were reported as mean and standard deviation (SD). Unpaired Student' *t*-test was used to explore the difference in the intercanine distance and mesiodistal canine width between males and females. The level of significance was set at 0.05. The statistical analysis was performed using the Statistical Package Software for Social Sciences (SPSS), version 18.0 (SPSS Inc., Chicago, IL, USA).

## 3. Results

The descriptive statistics and comparison between the two genders are shown in Table 1: the right canine width, left canine width, and intercanine distance were significantly greater in males compared to females (*P* < 0.0001). The mean intercanine distance was 27.624 ± 1.59 mm in males and 25.927 ± 1.226 mm in females. The mean widths of the right and the left canines in males were 7.188 ± 0.314 mm and 7.187 ± 0.350 mm compared to 6.549 ± 0.332 mm and 6.541 ± 0.343 mm in females, respectively.

The left canine was found to reveal greater sexual dimorphism (9.9%) as compared with right canine (9.7%). However, the right and left canine widths were not found to be statistically different within each gender (Table 2).

The 95% confidence interval of canine width and intercanine distance for the group of Lebanese adults is presented in Table 3. It shows that there is a probability of 95% that the mean size of the right and left mandibular canines is greater than 7.104 and 7.094 mm, respectively, in males and lower than 6.627 and 6.622 mm, respectively, in females.

## 4. Discussion

The present study establishes the morphometric criteria of the canine size and recognizes a significant sexual dimorphism in mandibular canines in a group of Lebanese adults aged between 18 and 25 years. It also indicates the probability of male sex determination to an extent of 95% when the width of the mandibular canine is greater than 7.104. However, the right and left canine widths were not statistically significant within males or females.

The age range chosen provides the best sample for tooth size measurements because early adulthood dentitions have less attrition in most individuals. Consequently the effect of these factors on the actual mesiodistal tooth width will be minimal especially on the mandibular canines that have a mean age of eruption of 10.87 years.

We chose to perform our measurements on casts as Hunter and Priest indicated since there was considerable advantage in the measurement of teeth on the dental cast rather than measuring teeth directly in the mouth [16]. Measurements on casts are straightforward and credible. However, Kaushal et al. demonstrated that measures conducted on casts were not significantly different from intraoral measures [18].

Sexual dimorphisms in mesiodistal tooth width had been of interest to several investigators [1–9]. A study conducted in Saudi Arabia on males and females aged between 13 and 20 years indicated that among all teeth, only the canines in both jaws revealed a significant sexual difference [19]. Similar findings were reported in a study on ethnic Chinese population with normal occlusions [20]. The results of our study are in agreement with a study conducted in other different populations: Indian [18], Nigerian [19], and Brazilian [20], demonstrating that mandibular canine width and intercanine distance were useful parameters in differentiating between the two genders. However, our study did not confirm that sexual dimorphism was great in left mandibular canine as demonstrated in many previous studies [21–23].

The present study also indicates the probability of sex determination to an extent as high as 95%: when the mean width of either canine is greater than 7.188 mm, the sex is male. This finding in the group of Lebanese adults is of definite significance as the determination of sex makes identification easier and this is of immense forensic importance. The study conducted in Indian population shows that when the width of canine is greater than 7 mm, the probability of the individual being a male is 100% [18].

Canine width and intercanine distance are of definite significance in determination of sex, and it is of immense forensic importance in a Lebanese population as well as many populations in which sexual dimorphism was proved [2–8, 16, 19–21]. Environmental factors and eating habits have been found to have effect on tooth size [22]. It was postulated that in the evolution of primates, the canines are functionally not masticatory and are related to threat of aggression and actual aggression. This aggressive function was dependent on canines especially in males. Therefore, in the present day humans, sexual dimorphism in mandibular canines is not only a coincidence but can be expected to be based on functional activity [24, 25]. Sexual dimorphism in canine size is influenced markedly by genetic factors. Both X and Y chromosomal involvement has been found by various authors [25].

Garn et al. had correlated sexual dimorphism in canines with stature, weight, bone age, menarche in girls, and the time of epiphysis union. These associations suggested direct influence of steroidal hormones on tooth development and maturation. They found that tooth eruption is accelerated in early maturing girls, indicating that steroid hormones of gonad and adrenal origin may be involved in the relationship between sexual maturation and dental development [24].

The parameters measured in the present study will be of great help in identification of sex in forensic investigations in the Lebanese population. Similar studies could be done on other populations in order to examine the applicability of this sexual dimorphism method in determining gender when partial human remains are recovered. It must be noted, however, that identification of sex through mandibular canine has its limitations; the gender of the subject to whom the fragment of the mandible belongs can be determined adequately when the fragment is found in the geographical area where the subject was born.

## 5. Conclusion

The present study demonstrates that mesiodistal width of the right and left canines as well as the intercanine distance was significantly greater in males. It also indicates the probability of male sex determination to an extent as high as 95% when the width of either canine is greater than 7.104 mm. It can be concluded that the measurement on mandibular canine is a quick and easy method for determining sex and in identification of an unknown individual in Lebanese population.

## Figures and Tables

**Table 1:** Comparison of intercanine distance and canine width between males and females.

Variables	Sex	*N *	Mean	SD	*P* value
Intercanine distance (mm)	Males	54	27.624	1.590	<0.0001
	Females	69	25.927	1.226	
Right canine width (mm)	Males	54	7.188	0.314	<0.0001
	Females	69	6.549	0.332	
Left canine width (mm)	Males	54	7.187	0.350	<0.0001
	Females	69	6.541	0.343	

**Table 2:** Comparison between right canine and left canine widths (mm) within each gender.

	Right canine width		Left canine width		*P* value
	Mean	SD	Mean	SD	
Males	7.188	0.314	7.187	0.350	0.996
Females	6.549	0.332	6.541	0.343	0.998

**Table 3:** 95% confidence interval of canine width and intercanine distance in males and females.

	Right canine width		Left canine width		Intercanine distance	
Males	7.104	7.272	7.094	7.280	27.200	28.048
Females	6.471	6.627	6.460	6.622	25.638	26.216
